# Salivary Level of Glutathione Reductase, Catalase, and Free Thiol in Patients With Temporomandibular Joint Disorder: A Cross‐Sectional Study

**DOI:** 10.1155/ijod/3051423

**Published:** 2026-09-14

**Authors:** Fahimeh Rezazadeh, Nima Fassihi, Dorsa Mahdavi, Sara Maroufi, Elham Tayebi Khorami, Amir Tabesh, Hossein Sedarat

**Affiliations:** ^1^ Oral and Dental Disease Research Center, Department of Oral and Maxillofacial Medicine, School of Dentistry, Shiraz University of Medical Sciences, Shiraz, Iran, sums.ac.ir; ^2^ Department of Oral and Maxillofacial Surgery, School of Dentistry, Shiraz University of Medical Sciences, Shiraz, Iran, sums.ac.ir; ^3^ Prosthodontic Department, School of Dentistry, Shiraz University of Medical Sciences, Shiraz, Iran, sums.ac.ir; ^4^ Student Research Committee, School of Dentistry, Shiraz University of Medical Sciences, Shiraz, Iran, sums.ac.ir; ^5^ Student Research Committee, Jahrom University of Medical Sciences, Jahrom, Iran, jums.ac.ir

**Keywords:** catalase, free thiol, glutathione reductase, saliva, temporomandibular joint disorder

## Abstract

**Objective:**

The role of antioxidants in temporomandibular joint dysfunction has been suggested; however, no studies have measured the saliva of patients with TMD. This study aimed to assess the concentrations of glutathione (GSH) reductase, catalase (CAT), and free thiol in the saliva of patients with TMD, compared with individuals without TMD.

**Materials and Methods:**

In this cross‐sectional study, 35 patients with TMD, who had at least one revised comprehensive diagnostic (RDC) criteria for TMD (pain in TMJ and masticatory muscles; muscle spasm or clicking), and 20 healthy individuals (without TMD) participated, and their saliva sample (5 mL) was collected in the morning. The disease severity was measured based on the Helkimo index. Salivary levels of GSH reductase (wavelength = 405 nm), CAT (wavelength = 520 nm), and free thiol (wavelength = 405 nm) were measured using enzyme‐linked immunosorbent assay (ELISA). Data were statistically analyzed.

**Results:**

Patients with TMD had significantly lower salivary levels of GSH reductase (253 mU/mL in the TMD group and 390 mU/mL in the control group), and CAT (159 mU/mL in the TMD group and 270 mU/mL in the control group) compared with the control group, but similar free thiol levels (60 mU/mL in the TMD group and 56 mU/mL in control group). There was a correlation between antioxidant status and the presence of click, pain, and severity of the disease; however, there was no correlation between age and sex with antioxidant contents.

**Conclusion:**

The results suggest that these antioxidants are affected by TMD and may be indicative of disease severity. Further studies can determine the diagnostic accuracy of these three antioxidants and their potential role to be used as diagnostic and prognostic biomarkers for monitoring and treating TMD.

**Clinical Relevance:**

TMD is common, and oxidative stress (OS) may play a role in their development. Using saliva as a diagnostic method offers a less invasive alternative to synovial fluid or blood.

## 1. Introduction

Temporomandibular joint disorders (TMDs) are pathological states involving muscular and/or articular and periarticular tissue structure and function, resulting in pain and discomfort and movement restriction [1]. As the disorder occurs in the face, these symptoms can also result in psychological stress and reduce the patient’s quality of life. The etiology of TMD is not well understood, and it is currently considered multifactorial, resulting from a complex of pathophysiological, psychological, and anatomical factors [2].

Oxidative stress (OS), the imbalance between the formation and uptake of free radicals, is the initiator of many inflammatory processes [3, 4]. Antioxidants counteract the toxicity of cytokines, free radicals, and reactive oxygen species (ROS), and their reduction results in OS [5], found to be involved in the pathophysiology of many diseases. Similarly, in TMD, the reduced enzymatic and nonenzymatic oxidative antioxidants have been suggested to establish the oxidative process, also related to the severity of the disease [6]; counteractively, TMJ disk derangement, degenerative changes, trauma, and mechanical stress may all result in OS [7].

The measurement of biomarkers has been suggested as an objective metric to investigate the validity of clinical investigations, including the component of musculoskeletal aches that would produced [8]. Biomarker values in the TMD patients were determined to explain pain mechanisms and enable early detection of joint pain and destruction to avoid the progression of pain and disability [3, 9].

Thiol groups are antioxidants that protect cells from OS, and measurement of free (sulfhydryl) thiol levels is suggested as a biomarker of OS [10], involved in the pathophysiology of oral and periodontal diseases [11]. Catalase (CAT) is a normal enzyme found in most beings exposed to oxygen, protecting the cells from oxidative harms. Glutathione (GSH) reductase catalyzes GSH disulfide (GSSG) to the sulfhydryl form, GSH, playing an essential role in OS and cell death. The GSSG/GSH ratio is suggested to indicate the cell’s oxidative stability, with higher levels indicating less OS [12]. The delicate balance to maintain low amounts of GSSG and large quantities of GSH is provided by GSH reductase [12], the measurement of which is suggested to indicate the level of OS in various diseases [13]. Also, patients with TMD have been found to have increased serum levels of CAT and GSH, compared to healthy controls [14], with higher concentrations documented in the synovial fluid of patients at higher disease stages [6]. Saliva is a protecting fluid of the oral cavity, an available liquid that can be obtained easily from patients for diagnostic purposes with less disturbance, compared to blood sample or synovial fluid, performed self‐administered without specific tools, personnel, or high costs. Considering the significant antioxidant structure of saliva, this liquid has been used in several studies for measurement of enzymatic and nonenzymatic antioxidants [15, 16]; also for identifying the main degenerative changes in the TMJ and the role of OS in this disease, including the cortisol salivary level, reflecting the stress response pathophysiology in TMD [17]. Furthermore, the concentrations of OS biomarkers in the synovial fluid of patients with TMD are found to be correlated with salivary levels [18]. Therefore, saliva seems an appropriate fluid for the measurement of OS biomarkers in patients with TMD, although other biomarkers of OS, like cortisol, have been measured in saliva of patients with TMD, indicating promising results [18]. However, previous studies have not measured the salivary level of GSH reductase, CAT, and free thiol in patients with TMD. Therefore, the present study aimed to measure the salivary level of GSH reductase, CAT, and free thiol in patients with TMDs, compared with the control group, and find out the association between these values and the disease severity.

## 2. Material and Methods

### 2.1. Study Design and Participants

In this cross‐sectional study (from 2019 to 2020), patients with TMD, who referred to the Oral and Maxillofacial Disease Department of Shiraz Dental Faculty, were considered as the case group, and age‐ and sex‐matched healthy individuals without TMD (who referred to the dental clinic in the same faculty) were considered as the control group. Diagnosis of TMD was based on having at least one revised comprehensive diagnostic (RDC) criteria for TMD (pain in TMJ and masticatory muscles; muscle spasm or clicking), and the disease severity was determined by clinical examination based on the Helkimo‐Index, including TMJ pain, muscle tenderness, jaw movement, click sound, and deviation [19].

The sample size was calculated as 16 participants per group based on a comparison of two independent means, assuming a two‐sided significance level of 0.05, a statistical power of 80%, a standard deviation of 0.1, and a minimum clinically significant difference of 0.1 between the two groups. To increase the statistical power, 35 patients were included in the case group and 20 in the control group. Participants were enrolled based on the inclusion criteria after providing written informed consent. Patients with other inflammatory disorders, those who had used anti‐inflammatory medications, muscle relaxants, analgesics, vitamin E or vitamin C in the past 2 months, current smokers, and those who had previously received treatment for TMD were excluded.

### 2.2. Data Collection and Saliva Sampling

An unstimulated saliva sample (5 mL) was collected from each participant in a sterile container in the morning, in accordance with the circadian rhythm, after 1 h of refraining from chewing gum, applying lipstick, consuming food, or consuming any liquids other than water. The morning was chosen as saliva flow is typically lower in the morning, which made it easier to collect a sample, and participants were less likely to have eaten or drunk anything in the morning, which reduced the risk of saliva contamination.

Participants were instructed to rinse their mouth for 30 s with clean water and discard the water and not to salivate or think or talk about food.

Samples were frozen at −20°C and stored until the start of the analysis. The salivary level of GSH reductase, CAT, and free thiol was measured by the enzyme‐linked immunosorbent assay (ELISA) technique using a Kiazist kit (Kiazist Co., Iran) according to its instructions. The samples were assessed via a microplate reader with an optical density (OD) range of 0–3 OD and at a wavelength of 405 nm for GSH reductase, 520 nm for CAT, and 405 nm for free thiol.

### 2.3. Data Analysis

Data were analyzed by SPSS software Version 25.0 (SPSS Inc., IL, USA), applying the mean ± SD and frequency (%). Independent sample *t*‐test and chi‐square test were used to compare data, including age, sex, pain severity, and the Helkimo index between groups. Meantime, a repeated measure ANOVA (RM‐ANOVA), was used to assess the changes in some measurements. *p*  < 0.05 was considered significant.

## 3. Results

Thirty‐five patients with TMD were compared with 20 healthy individuals without TMD (there were no missing cases). The total mean age of the study population was 31.38 ± 10.55 (range: 19–65) years without difference between the groups (33.77 ± 11.88 years in the case group vs. 31.85 ± 5.98 years in the control group; *p* = 0.43). In total, 36 women and 19 men were included; 11 women and nine men in the control group and 25 women and 10 men in the TMD group (*p* = 0.21, based on the results of chi‐square test).

Among patients with TMD, 16 patients expressed pain (45.7%), 11 patients had left click (31.4%), seven had right click (20%), 11 had bilateral click (31.4%), and six patients had no sign of click (17.1%); nine patients had left deviation (25.7%), nine had right deviation (25.7%), and 17 patients had no sign of deviation (48.6%).

The mean salivary level of GSH reductase was lower in the case group (253 mU/mL) compared to the control group (390 mU/mL), and the mean CAT level was also lower in the case group (159 mU/mL) compared to the control group (270 mU/mL) (*p* = 0.001). However, no significant difference was observed in free thiol levels between the two groups (60 mU/mL in the TMD group vs. 56 mU/mL in controls; *p* = 0.75), based on the results of independent sample *t*‐test.

For evaluating the effect of age and sex on the antioxidant levels, we compared the levels of GSH reductase, CAT, and free thiol based on age or sex, the results of which showed no significance (*p*  > 0.05; Table 1).

**Table 1:** Comparing the antioxidant levels based on the participants’ age and sex.

Type of antioxidants (mU/mL)	Variable	*p*‐Value
Glutathion reductase	Age Sex	0.543^a^ 0.142^∗∗^

Catalase	Age Sex	0.952^a^ 0.489^∗∗^

Free thiol	Age Sex	0.257^a^ 0.201^∗∗^

*Note*: *p*‐values were calculated using one‐way ANOVA for age and the chi‐square test for sex.

^a^All antioxidant levels are presented in mU/mL (milliunits per milliliter).

^∗∗^ indicates independent‐samples *t*‐test.

In addition to comparing the case group with the control group, we also performed subgroup analysis in the case group to indicate the effect of the disease severity on the levels of antioxidants, and the results showed lower levels of GSH reductase (*p* = 0.001) and CAT (*p* = 0.020) in patients suffering from pain, compared to those without (Table 2), lower levels of GSH reductase (*p* = 0.001) and CAT (*p* = 0.001) in those with click, compared to those without (Table 3), but no significant difference in the mean level of any of the three antioxidants between patients with or without deviation (*p*  > 0.05; Table 4).

**Table 2:** Comparing antioxidant levels based on the presence of pain in the case group.

Type of antioxidant	Pain presence	Antioxidant level (mU/mL)	*p*‐Value
Glutathion reductase	Yes (*N* = 16) No (*N* = 19)	154.4 ± 128.2 337.1 ± 138.5	<0.001

Catalase	Yes (*N* = 16) No (*N* = 19)	129.6 ± 54.6 183.7 ± 75.2	0.020

Free thiol	Yes (*N* = 16) No (*N* = 19)	57.8 ± 20.9 62.6 ± 65.5	0.782

*Note:* Values are presented as mean ± SD (mU/mL). *p*‐values were obtained using the independent samples *t*‐test.

Abbreviation: mU/mL, milliunits per milliliter.

**Table 3:** Comparing antioxidant levels based on the presence of click in the case group.

Type of antioxidant	Click	Antioxidant level (mU/mL)	*p*‐Value
Glutathion reductase	Yes (*N* = 30) No (*N* = 5)	224.3 ± 154.3 429.0 ± 53.8	<0.001

Catalase	Yes (*N* = 30) No (*N* = 5)	139.1 ± 53.4 278.3 ± 39.0	<0.001

Free thiol	Yes (*N* = 30) No (*N* = 5)	63.3 ± 52.9 43.5 ± 16.9	0.419

*Note:* Values are presented as mean ± SD (mU/mL). *p*‐values were obtained using the independent samples *t*‐test.

Abbreviation: mU/mL, milliunits per milliliter.

**Table 4:** Comparing antioxidant levels based on the presence of jaw deviation in the case group.

Type of antioxidant	Deviation	Antioxidant level (mU/mL)	*p*‐Value ^∗^
Glutathion reductase	Yes (*N* = 18) No (*N* = 17)	216.7 ± 155.2 292.6 ± 162.4	0.167

Catalase	Yes (*N* = 18) No (*N* = 17)	142.5 ± 55.4 176.4 ± 82.8	0.162

Free thiol	Yes (*N* = 18) No (*N* = 17)	75.0 ± 65.5 45.0 ± 14.2	0.075

*Note:* Values are presented as mean + SD (mU/mL).

Abbreviation: mU/mL, milliunits per milliliter.

^∗^ *p*‐values were obtained using the independent samples *t*‐test.

Also, the results showed lower levels of GSH reductase (*p* = 0.001) and CAT (*p* = 0.011) in individuals with more than 10 points on the Helkimo index, compared to those with less than 10 scores (Table 5) among patients with TMD.

**Table 5:** Comparison of antioxidant levels based on the patient’s score of the Helkimo index in the case group.

Type of antioxidant	Number of points on Helkimo index	Antioxidant level (mU/mL)	*p*‐Value ^∗^
Glutathion reductase	<10 (*N* = 18) ≥10 (*N* = 17)	378.5 ± 114.6 121.3 ± 71.1	<0.001

Catalase	<10 (*N* = 18) ≥10 (*N* = 17)	187.9 ± 71.9 128.3 ± 57.5	0.011

Free thiol	<10 (*N* = 18) ≥10 (*N* = 17)	67.1 ± 67.4 53.4 ± 18.1	0.425

*Note:* Values are presented as mean + SD (mU/mL).

Abbreviation: mU/mL, milliunits per milliliter.

^∗^ *p*‐values were obtained using the independent samples *t*‐test.

## 4. Discussion

The results of the present study showed that patients with TMD had lower salivary levels of GSH reductase and CAT compared with the matched controls. Furthermore, reduced levels of these two biomarkers were observed among patients with severe symptoms, including pain and clicking, as well as in those with greater disease severity according to the Helkimo Index, suggesting that these antioxidants are affected by TMD. These results suggest these two biomarkers as valuable markers for confirming the presence and severity of TMD among patients and confirmed our primary hypothesis on the association of the salivary levels of the selected antioxidants (GSH reductase, CAT, and free thiol) with the presence and severity of TMD, except for thiol, on which no significance was found. Supposedly, this association can be related to the formation of excess free radicals in tissue as a result of the ROS‐induced mechanical stress that leads to this pathological condition, TMD, stimulating excessive biological processes, including DNA damage, lipid oxidation, protein denaturation, and tissue harm, deteriorating inflammation in patients with TMD [20]. Ascorbate and GSH are two low‐molecular‐weight antioxidants (LMWA), able to scavenge free radicals and activate antioxidant defense system to inhibit free radical reactions and oxidative damage [21]. However, the present study was performed in the clinical phase, and further studies are required to determine the exact mechanism of these biomarkers in patients with TMD.

As far as we are concerned, no previous study has assessed the salivary level of these antioxidants in patients with TMD; nonetheless, these results are in line with various research on OS markers salivary samples of patients with TMD, discussed below, though measuring different biomarkers. The study by AlSahman et al. [17] showed higher salivary levels of cortisol in patients with TMD compared with healthy controls, which is consistent with the results of the present study, considering the association of stress biomarkers with TMD, although the type of biomarker selected differed. They also pointed out the value of obtaining a sample early in the morning (vs. late morning) and higher levels in patients with disc displacement without reduction with limited mouth opening, which might be specific to cortisol. Lawaf et al. [22] measured total antioxidant capacity (TAC) levels in saliva and serum of patients with TMD using spectrophotometrical index and documented no association between the salivary and plasma levels of TAC; while they documented significant difference among patients with/without pain and the control group, there was no significant difference in salivary TAC levels. These results are contrary to that obtained in the present study, which might be due to the different biomarkers selected. In a study by de Almeida and Amenábar [23] on the changes in the salivary oxidative status of patients with TMD suffering from pain, the researchers perceived a higher OS index in these patients with significantly reduced TAC (determined through Erel’s method); thereby, documenting the role of oxidative changes in the pathogenesis of pain in patients with TMD. Their findings are consistent with the present results, which indicate that individuals who experience pain have lower antioxidant values.

Other studies have considered the serum or synovial levels of antioxidants, rather than that from saliva, and investigated their association with TMD and its severity. Demir et al. [14] surveyed the serum levels of malondialdehyde (MDA), an OS indicator, and three antioxidant enzymes, including GSH, CAT, and superoxide dismutase (SOD), in patients with TMD, compared with the control group, and concluded that OS indicators might be promising biomarkers for diagnostic and therapeutic goals in TMJ disorders, consistent with that provided in the present study, considering CAT and GSH. Also, in a study of Fleifel and AlKhiary [24], OS biomarkers, including SOD, MDA, and GSH, were measured in synovial fluid of 10 patients with TMD, compared with 6 healthy individuals; the results showed increased SOD and MDA in patients, while GSH decreased. Their findings support the potential role of antioxidant agents in the management of TMJ‐related pain and dysfunction, which is in agreement with the results of the present study. However, they reported no significant association for GSH, in contrast to our findings [24]. Other researchers have also found no association between TMD and GSH in patients with TMJ internal derangement, compared with controls, while suggesting higher serum prolidase activity and OS in patients vs. healthy individuals [25]. These results are contrary to the results of the current study on the GSH level, which might be because they have included a subgroup of patients with TMD, namely, patients with internal derangement, or because serum was used rather than saliva.

Regarding disease severity, most studies have reported a significant association with OS biomarkers, which is consistent with the findings of the present study. However, these investigations have primarily focused on synovial tissue rather than saliva [26]. Measuring SOD activity in the synovial fluid of patients with internal derangement of TMJ using the nitrobluetetrazolium (NBTH2) reduction rate demonstrated the correlation of disease severity (Helkimo index and VAS) with the level and activity of antioxidants [27]. Also, radiological evaluation of intra‐articular structures in patients with TMD showed the correlation between the OS in synovial fluid and closed lock in these patients [28]. These findings are consistent with those of the present study, which demonstrated reduced salivary antioxidant capacity in patients presenting with pain and clicking, although the biological sample analyzed differed between the studies. Given the limited evidence based on salivary samples, further research is warranted to establish definitive conclusions regarding the association of GSH reductase and CAT levels with TMD, its severity, and related clinical symptoms.

Considering the levels of free thiol, we found no significant difference between the case and control groups nor based on the disease symptoms and severity. Other studies have also documented thiol change in other diseases, like the decreased levels documented in the gingival tissue of patients with gingivitis and periodontitis [11], but none have considered TMD to be comparable to our results. Also, salivary protein thiol was significantly higher in patients with brain tumor, compared with healthy participants (measured by the spectrophotometric method using dithionitrobenzene DTNB‐Ellman’s method) [29]. Nonetheless, the condition of those patients differed from that of the current study. The lack of significant change in the thiol level in the present study may be associated with the reduced form of intracellular GSH, counterbalanced by an alternative compensatory pathway involving extracellular free thiol protein, thereby maintaining a constant level of measurable free thiol in the present study.

Based on the results of the current study, age and sex did not influence the antioxidant levels. In accordance with these results, Lawaf et al. [22] also showed no association between age and sex of patients with plasma TAC levels. Moreover, Demir et al. [14] showed that the expressive features (sex and age) did not affect the serum levels of MDA, GSH, CAT, and SOD in patients with TMJ disorders or in the control group. In contrast, AlSahman et al. [17] found significantly higher cortisol levels in males compared with females. This sex‐related difference may be specific to cortisol as no significant differences between males and females were detected for the biomarkers assessed in the present study [17]. Further studies are needed to clarify the role of sex in the relationship between OS biomarkers and the presence and severity of TMD.

The limitations of the present study include the small sample size, lack of randomization, and lack of access to an accredited laboratory to achieve the highest reliability for saliva test results. Therefore, we incidentally evaluated various laboratories and several kits to include those with the highest reliability and validity among the ones available. Another important limitation that should be acknowledged is the risk of the effect of confounders, which we have not evaluated in the present study, like stress, bruxism, and parafunctional habits, on the results of the study. Furthermore, this study was performed as a single center cross‐sectional study; therefore, the study population may not represent the whole population in Iran, and further studies are required for generalizability of results to the whole population. Also, it was better to select the control group from the general population, rather than people referring to the dental clinic, for a broader perspective.

## 5. Conclusion

In the current study, it was concluded that the salivary levels of GSH reductase and CAT can be used as an easily‐accessible measurement tool to help diagnose TMD and estimate its severity and symptoms (clicking and pain), irrespective of the patient’s age and sex. Future studies, considering the limitations of the present study, can suggest the diagnostic accuracy of salivary GSH reductase and CAT, which might result in suggesting them as a diagnostic biomarker for TMD monitoring and treatment progress.

**Nomenclature**
TMJ: Temporomandibular joint
TMD: Temporomandibular joint disorders
OS: Oxidative stress
CAT: Catalase
ROS: Reactive oxygen species
GSSG: Glutathione disulfide
GSH: Glutathione, the sulfhydryl form
RDC: Revised comprehensive diagnostic
ELISA: Enzyme‐linked immunosorbent assay
OD: Optical density
LMWA: Low‐molecular weight antioxidants
SOD: Superoxide dismutase
NBTH2: Nitrobluetetrazolium
TAC: Total antioxidant capacity
DTNB: Dithionitrobenzene
MDA: Malondialdehyde
VAS: Visual analog scale
TOS: Total oxidant status
TMJ‐ID: Temporomandibular joint internal derangement.

## Author Contributions

Fahimeh Rezazadeh: conceptualization, formal Analysis, methodology, writing – original draft preparation, writing – review and editing. Nima Fassihi: conceptualization, data curation, investigation, writing – original draft preparation, writing – review and editing. Dorsa Mahdavi, Sara Maroufi, Elham Tayebi Khorami, Amir Tabesh, and Hossein Sedarat: formal analysis, methodology, writing – original draft preparation, writing – Review & Editing.

## Funding

This research did not receive any specific grant from funding agencies in the public, commercial, or not‐for‐profit sectors.

## Disclosure

All authors have read and approved the final version of the manuscript and agree to be accountable for all aspects of the work.

## Ethics Statement

This study was approved by the Ethics Committee of Shiraz University of Medical Science (Code: IR.SUMS.DENTAL.REC.1398.108). Written informed consent was obtained from all participants involved in the study.

## Conflicts of Interest

The authors declare no conflicts of interest.

## Data Availability

The final data used to support the findings of this study are included within the article. Furthermore, the primary data used to support the findings of this study are restricted by the Ethics committee of Shiraz University of Medical Sciences in order to protect patient privacy. Data are available from corresponding author for researchers who meet the criteria for access to confidential data.
